# tcR: an R package for T cell receptor repertoire advanced data analysis

**DOI:** 10.1186/s12859-015-0613-1

**Published:** 2015-05-28

**Authors:** Vadim I. Nazarov, Mikhail V. Pogorelyy, Ekaterina A. Komech, Ivan V. Zvyagin, Dmitry A. Bolotin, Mikhail Shugay, Dmitry M. Chudakov, Yury B. Lebedev, Ilgar Z. Mamedov

**Affiliations:** Shemyakin-Ovchinnikov Institute of Bioorganic Chemistry, 16/10 Miklukho-Maklaya, Moscow, 117997 Russia; National Research University Higher School of Economics, 20 Myasnitskaya Ulitsa, Moscow, 101000 Russia; Central European Institute of Technology, Masaryk University, Brno, Czech Republic

**Keywords:** Adaptive immunity, T cell receptor, TR repertoire analysis, TR diversity

## Abstract

**Background:**

The Immunoglobulins (IG) and the T cell receptors (TR) play the key role in antigen recognition during the adaptive immune response. Recent progress in next-generation sequencing technologies has provided an opportunity for the deep T cell receptor repertoire profiling. However, a specialised software is required for the rational analysis of massive data generated by next-generation sequencing.

**Results:**

Here we introduce tcR, a new R package, representing a platform for the advanced analysis of T cell receptor repertoires, which includes diversity measures, shared T cell receptor sequences identification, gene usage statistics computation and other widely used methods. The tool has proven its utility in recent research studies.

**Conclusions:**

tcR is an R package for the advanced analysis of T cell receptor repertoires after primary TR sequences extraction from raw sequencing reads. The stable version can be directly installed from The Comprehensive R Archive Network (http://cran.r-project.org/mirrors.html). The source code and development version are available at tcR GitHub (http://imminfo.github.io/tcr/) along with the full documentation and typical usage examples.

## Background

The power of the human adaptive immunity is realised throughout the immunoglobulins (IG) and T cell receptors (TR): the highly diverse antigen receptors which recognise pathogens and provide specific immune responses. Until recently, studies on the structural composition of immune repertoires, receptor sequence sharing and quantitative estimation of particular B or T cell clones abundance have remained a challenge due to an extremely high diversity of IG and TR sequences: the maximal theoretical diversity of the most variable TR beta chains is estimated as 1 × 10^14^ [1] and 1 × 10^18^ for the heterodimeric T cell receptor consisting of α and β chains [2–4].

Next-generation sequencing (NGS) technologies have opened a new era in the field of IG and TR repertoires research, which includes the studies on adaptive immune system ageing [5], immune repertoire reconstitution after therapy [6], response to vaccines [7] and subpopulation repertoire structure [8, 9]. In addition to standard IMGT/HighV-QUEST [10–12] recent studies provided powerful tools for processing raw IG/TR NGS data: extraction of complementarity determining regions (CDR) from reads and generation of clonotype (hereafter clonotype is a group of sequencing reads with identical aminoacid or nucleotide CDR3 sequence and V/J genes) sets [12–18], as well as advanced algorithms for the correction of PCR and sequencing errors [19, 20]. However, the interpretation of TR repertoires (i.e., lists of TR clonotypes with their quantities) in terms of biological relevance requires further downstream analysis of the resultant clonotype sets.

In order to examine TR repertoires of different individuals a number of strategies can be employed such as quantifying the number of shared nucleotide and amino acid sequences between repertoires, comparisons of gene usage frequencies and repertoire diversity estimation [21]. Only two software tools that apply a limited number of the analysis methods - MiTCRViewer [13] and ViDJiL [15] are available.

Here, we introduce tcR: an R package for the analysis of TR repertoires that integrates widely used methods for individual repertoires analyses and TR repertoires comparison: gene usage comparison, customisable search for clonotypes shared among repertoires, spectratyping, random TR repertoire generation, various repertoire diversity measures and other commonly used approaches to the repertoire analysis.

### Implementation

This section describes the input data format, methods and procedures implemented in tcR. The R package vignette presents a more detailed overview of methods included in tcR.

1. **Input data and data manipulation:** The input data for tcR are tab-delimited files with rows representing clonotypes and columns representing read counts, nucleotide and amino acid sequences of the CDR3, names and borders of the identified V(ariable), D(iversity) and J(oining) genes and the number of insertions at gene junctions. This file format is a default output of the MiTCR software [13] that is widely used for TR NGS data extraction and raw clonotype set generation (see the package vignette for the detailed information on valid input file formats). TR repertoires are represented in tcR as R data frames, therefore they could be easily assigned to subsets, filtered and transformed using basic and effective R subroutines.
2. **Descriptive statistics:** The tcR package provides utilities for computing primary descriptive statistics for TR repertoires, including, but not limited to, counts and percentages of TR nucleotide or amino acid clonotypes, V and J gene usage, clonal count skewness and distribution of CDR3 sequence lengths.
3. **Shared clonotypes analysis and repertoire comparison:** The tcR applies a diverse set of intersection procedures and a set of similarity measures to the compared repertoires: intersection by nucleotide or amino acid CDR3 sequences, Jaccard index, Morisita’s overlap index and sequential intersection of the most abundant clonotypes among repertoires (”top cross”, i.e. intersection between top-1000 from one repertoire with top-1000 from the other, then between top-2000 clonotypes, etc., see Fig. 1c).

**Fig. 1:**
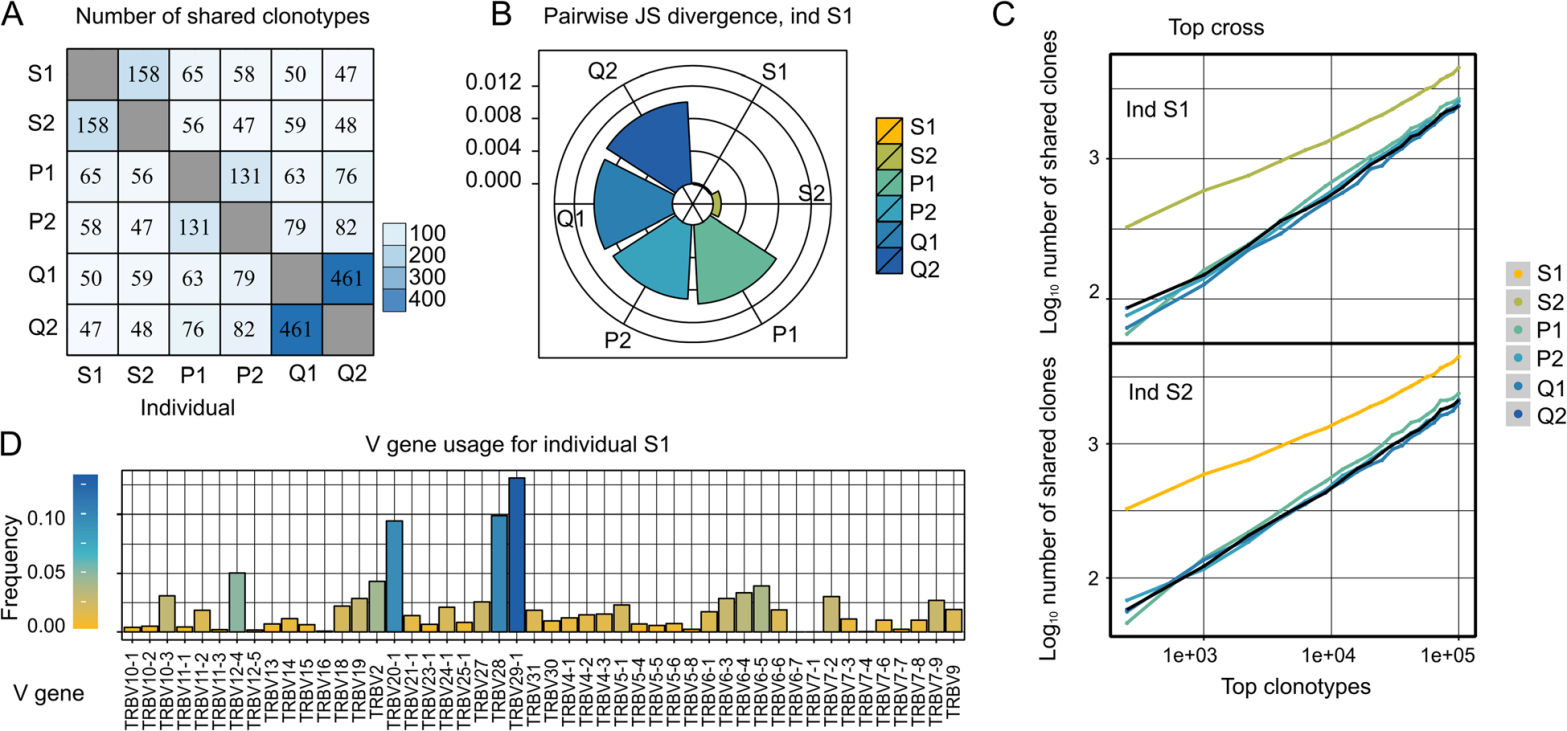
Examples of tcR data analysis visualisation. **a**. Heatmap of number of clonotypes with identical CDR3 amino acid sequences shared among individuals. **b**. Jensen-Shannon divergence of V gene usage for individual S1 and other individuals. **c**. The normalised number of identical TRB CDR3 nucleotide sequences in the top 1000 most abundant clonotypes, 2000 most abundant clonotypes, etc. for each possible pair of individuals. **d**. V gene usage for individual S1
4. **Repertoire diversity and gene usage analysis:** For the analysis of the V and J gene usage, the package employs Shannon entropy measure, Jensen-Shannon divergence and Principal Component Analysis. To evaluate the repertoire diversity, the effective number of types (“true diversity”), Gini and Gini-Simpson indices, inverse Simpson index, Chao1 index and rarefaction analysis were implemented.
5. **Visualisation procedures:** The package provides a number of functions for generating plots, including heatmaps of the number of shared CDR3 sequences, (see Fig. 1a), histograms of V and J gene usage (see Fig.1d), radar bar plots of the Jensen-Shannon divergence of the V gene usage among individuals (see Fig.1b) and TR length spectratyping.
6. **Artificial repertoire generation:** The tcR package incorporates a procedure for artificial TR repertoire generation. Generative model provided with the package has been adopted from [1].

## Results and discussion

The rapidly increasing number of B and T cells high-throughput sequencing studies has led to the development of specialised programs able to manipulate with the specific IG and TR sequencing data [12–18]. The downstream analysis of generated clonotype sets is usually performed by researchers in each lab individually using various algorithms generating diverse results and conclusions. Here we made an attempt to collect the most widely used downstream processing applications in a single package to simplify the immune receptors data analysis. The R package tcR is mainly dedicated to two types of users. First, it can be utilised by the beginners in TR repertoire data analysis. We provide two alternative pipelines with automatic report generation similar to that described in [22] for the analysis of either a single repertoire or a group of repertoires. Second, it is suitable for needs of advanced users. With the power and flexibility of the R language and the tcR platform providing the common subroutines a user can easily employ his own analysis methods and concentrate on research without spending time on reinventing the wheel.

The existing software is mainly tailored to perform the first stage of the TR data analysis – CDR3 sequence extraction and clonotype sets generation. Some of these programs include several simple options for downstream data processing. However this set is limited to primary descriptive statistics and restricted visualization (see Table 1 for the detailed comparison). Thus we believe that our tcR package will be useful for the researchers working with adaptive immune repertoires data. The development of such software could result in standardisation of data analysis making possible the proper interpretation of results obtained by different groups worldwide.

**Table 1:** Comparison of tcR package with other existing TR data analysis software

Subroutines\Software	MiTCRViewer	ViDJiL	tcR
CDR3 sequence extraction and clonotype set building	+ (using MiTCR)	+	-
Sequence motif search	+	−/+ (exact match only)	+
Descriptive statistics (number of reads, number of clonotype sets, gene usage)	+	+	+
Number of shared clonotypes counting (with identical nucleotide or amino acid sequence)	-	-	+
Repertoire similarity measures (Jaccard index, Morisita’s overlap index)	-	-	+
Construction of shared clonotypes repertoires	-	-	+
Jensen-Shannon divergence computing for analysis of gene usage	-	-	+
PCA for analysis of gene usage	-	-	+
Repertoire diversity analysis (diversity estimation, rarefaction analysis, etc.)	-	-	+
Clonal abundance visualisation	-	+	+
Histograms of gene distributions	-	+	+
CDR3 length distribution visualisation	-	+	+
Radar plots for visualisation of distribution similarity (e.g., Jensen-Shannon divergence among set of distributions of genes)	-	-	+
Heatmaps for visualisation of repertoire similarity (e.g., number of shared clonotypes)	-	-	+
In silico spectratyping	+	+	+
Automatic report generation	-	-	+
Artificial repertoire generation	-	-	+

### Case studies

tcR was applied to TR alpha (TRA) and TR beta (TRB) repertoires analysis in our recent papers. In [22] we have shown distinctive properties of identical twins repertoires such as higher similarity of V and J gene usage (see Fig.1b, Jensen-Shannon divergence for twin pair S1-S2) and an increased number of in-frame and out-of-frame CDR3 sequences shared among the most abundant clonotypes (see Fig.1c). In [23] we demonstrate that mother and child have an increased portion of shared clonotypes with identical V genes in the shared clonotypes pool. As this study was applied to the previously obtained data no ethic committee conclusion was required.

### Future directions

In future we plan to continue adding new methods of repertoire comparisons, diversity estimation, to add parsers for data generated by Decombinator, IgBLAST and other software for extracting CDR3 sequences and aligning genes, and to optimise the package to efficiently handle large volumes of TR data for the analysis of massive repertoire data (tens of repertoires with millions of clonotypes). We plan to add more options for the automatic report generation, e.g. for tracking TR repertoires dynamics.

## Conclusions

The R package tcR is a platform designed for the analysis of TR repertoire data, which has two major advantages. First, it consolidates a wide spectrum of possible approaches to TR repertoire analysis in a single package. Second, it offers new methods for the comparative analysis of TR repertoires. The package has been applied to the experimental NGS data and allowed to obtain valuable observations of the inter-individual similarity of TR repertoires. The developed package is providing a wide range of new opportunities for the TR repertoire data analysis.

## Availability and requirements

**Project name:** tcR

**Project home page:**http://imminfo.github.io/tcr/

**Operating system(s):** Platform independent

**Programming language:** R [24], C++

**Other requirements:** R packages data.table, dplyr, ggplot2, grid, gridExtra, gtable, igraph, Rcpp, reshape2, roxygen2, stringdist, utils.

**License:** Apache v2.0 License

**Any restrictions to use by non-academics:** None

## Abbreviations

IG: Immunoglobulin
TR: T cell receptor
CDR: Complementarity determining regions
V: Variable
J: Joining
D: Diversity
PCA: Principal component analysis
